# Genome-Wide Mapping Defines a Role for C/EBPβ and c-Jun in Non-Canonical Cyclic AMP Signalling

**DOI:** 10.3390/cells8101253

**Published:** 2019-10-14

**Authors:** Jolanta Wiejak, Boy van Basten, Graham Hamilton, Stephen J. Yarwood

**Affiliations:** 1 Institute of Biological Chemistry, Biophysics and Bioengineering, School of Engineering and Physical Sciences, Heriot-Watt University, Edinburgh Campus, Edinburgh EH14 4AS, UK; jol03@onet.eu (J.W.); bv9@hw.ac.uk (B.v.B.); 2 Glasgow Polyomics, Wolfson Wohl Cancer Research Centre, Garscube Campus, University of Glasgow, Bearsden G61 1QH, UK; Graham.Hamilton@glasgow.ac.uk

**Keywords:** vascular endothelial cells, cyclic AMP, EPAC1, c-Jun, C/EBPβ transcriptome, chromatin, cell adhesion molecules

## Abstract

The novel exchange protein activated by cyclic AMP (EPAC1) activator, I942, induces expression of the suppressor of cytokine signalling 3 (SOCS3) gene, thereby inhibiting interleukin 6 (IL6) inflammatory processes in human umbilical vein endothelial cells (HUVECs). Here we use RNA-SEQ and ChIP-SEQ to determine global gene responses to I942, in comparison with cyclic AMP production promoted by forskolin and rolipram (F/R). We found that I942 promoted significant changes in the RNA expression of 1413 genes, largely associated with microtubule stability and cell cycle progression, whereas F/R regulated 197 genes linked to endothelial cell function, including chemokine production and platelet aggregation. A further 108 genes were regulated by both treatments, including endothelial regulatory genes involved in purinergic signalling and cell junction organization. ChIP-SEQ demonstrated that F/R induced genome-wide recruitment of C/EBPβ and c-Jun transcription factors, whereas I942 promoted recruitment of c-Jun to genes associated with IL6 signalling, with little effect on C/EBPβ activation. Despite this, certain key inflammatory genes, including IL6, VEGF, CCL2/MCP1, VCAM1, SELE and ICAM1 were regulated by I942 without significant c-Jun recruitment, suggesting an additional, indirect mode of action for I942. In this regard, SOCS3 induction by I942 was found to require c-Jun and was associated with suppression of IL6-promoted ERK MAP kinase and AKT activity and induction of ICAM1. Pharmacological inhibition of ERK and AKT also potentiated ICAM1 induction by I942. We therefore propose that c-Jun activation by I942 regulates endothelial gene expression in HUVECs through direct mechanisms, involving recruitment of c-Jun or, as for ICAM1, through indirect regulation of tertiary regulators, including SOCS3.

## 1. Introduction

Production of cyclic AMP in response to Gs-protein coupled receptor (GsPCR) activation leads to the regulation of a striking number of physiological processes, including control of metabolism, neuronal activity and immune cell processes through alterations in gene expression patterns in target cells [1]. Classically, positive regulation of transcription by cyclic AMP occurs through phosphorylation of members of the cyclic AMP response element (CRE)-binding protein (CREB) family of transcription factors by protein kinase A (PKA), leading to their nuclear translocation, interaction with conserved cyclic AMP response elements (CRE; TGACGTCA) and recruitment of transcriptional co-activators to target gene promoters [2]. The CREB family of activators functions in diverse physiological processes, including the control of cellular metabolism and growth-factor-dependent cell survival. In addition to CREB activation, cyclic AMP and PKA can suppress gene expression through cross-talk with non-GsPCR receptors, for example inhibition of ERK (mitogen-activated protein (MAP) kinase) activation by growth factor receptors [3].

Since the discovery of these gene-regulatory mechanisms, it has become clear that cyclic AMP now regulates a range of “non-canonical” gene-regulatory mechanisms that do not involve PKA. For example, elevations in intracellular cyclic AMP in response to activation of adenosine and prostaglandin GsPCRs in human umbilical vein endothelial cells (HUVECs) leads to inhibition of interleukin 6 (IL6) receptor activation of ERK and STAT3 [4]. This inhibition occurs independently of the classical route for cyclic AMP signalling, through PKA and CREB family members, but is rather dependent on the induction of the suppressor of cytokine signalling 3 (SOCS3) gene in response to activation of exchange protein activated by cyclic AMP (EPAC) 1 [4]. SOCS3 is an E3 ubiquitin ligase component that targets IL6 signalling components for proteolytic degradation [5], whereas EPAC1 is a specific guanine nucleotide exchange factor (GEF) for the Ras GTPase homologues Rap1 and Rap2 [6]. Cyclic AMP binding sites in EPAC proteins facilitate their direct activation by cyclic AMP, thereby relieving auto-inhibitory influences of the cyclic nucleotide binding domain (CNBD) toward the catalytic GEF domain [6]. Recent research now implicates EPAC1 in the regulation of multiple inflammatory processes in vascular endothelial cells, like HUVECs, including regulation of endothelial cell–cell junction stability and activation of integrins, reduction in endothelial permeability and down-regulation of IL6-mediated inflammatory processes [7].

Induction of SOCS3 by EPAC1 therefore supports a paradigm for CREB-independent gene induction in VECs, which we have shown to involve cooperativity between c-Jun and CCAAT enhancer binding protein (C/EBP) transcription factors, which bind to a key AP1 (consensus ATGAGTCAT) binding site in the SOCS3 promoter [4,8,9]. In this regard, our initial findings demonstrated mobilisation of C/EBPβ, which is sufficient to induce SOCS3 gene expression in HUVECs following EPAC1 activation [10]. However, maximal SOCS3 gene expression by cyclic AMP also requires supporting activity from the MAP kinases, ERK and JNK [11,12,13]. In this case ERK is activated by cyclic AMP, independently of both PKA and EPAC1 [11], and this is required for the phosphorylation and activation of multiple transcription factors associated with the SOCS3 promoter, including C/EBPβ (Thr235), STAT3 (Ser727) and SP3 (Ser73) [12]. JNK activation is also promoted by cyclic AMP and principally leads to activation of the AP1 transcriptional complex component c-Jun through phosphorylation on Ser63 [13]. Consistent with reports implicating EPAC proteins as regulators of JNK activity in diverse cell types [14,15], we previously found that activation of the SOCS3 minimal promoter by EPAC1 requires a single AP1 site that constitutively binds phosphorylated (Ser63) c-Jun in DNA-pull-down assays. c-Jun (Ser63) becomes further phosphorylated following cyclic AMP stimulation and specific activation of PKA, but not through selective activation of EPAC1. AP1 activation and SOCS3 induction by EPAC1 in HUVECs therefore occur independently of JNK activation and c-Jun phosphorylation on Ser63.

It is clear therefore, that non-canonical cyclic AMP-regulation of gene expression requires further investigation. In particular, we need to determine to what extent C/EBPs and c-Jun are required for the regulation of global gene expression by cyclic AMP in VECs. To begin to address this we have begun to develop potent, small molecule regulators of EPAC1 activity to determine its role in the control of inflammatory gene expression. The first of these, I942, was initially identified by high-throughput screening using the CNBD of EPAC1 as the target in a competition assay involving fluorescent cyclic AMP [16]. Subsequent analysis using nuclear magnetic resonance (NMR) revealed that I942 directly interacts with the EPAC1 CNBD and in vitro GEF assays revealed that I942 exerts agonist properties towards EPAC1 and very little agonist action towards PKA [16]. Here we aim to build on these studies to compare and contrast non-canonical transcriptional regulation by cyclic AMP and I942. To do this we use a combination of RNA-SEQ and ChIP-SEQ approaches to determine the extent to which cyclic AMP-elevation and I942 promote genome recruitment of c-Jun and C/EBPβ and the nature of the genes regulated by these treatments.

## 2. Materials and Methods

### 2.1. Materials

Pooled human umbilical vascular endothelial cells (HUVECs) and endothelial cell growth medium 2 (EGM2) were purchased from PromoCell (Heidelberg, Germany). Antibodies to ICAM1 and GAPDH were obtained from New England Biolabs UK Ltd. (Hertfordshire, UK). Anti-SOCS3 antibodies were purchased from Santa Cruz Biotechnology (Santa Cruz, CA, USA). SuperSignal™ West Pico Chemiluminescent Substrate was from Fisher Scientific (Loughborough, UK). Secondary antibodies, anti-rabbit-IgG horseradish peroxidase, anti-goat-IgG horseradish peroxidase and anti-mouse-IgG horseradish peroxidase conjugates, were from Sigma-Aldrich Company Ltd. (Dorset, England). Forskolin, rolipram and *N*-benzoyloxycarbonyl (*Z*)-Leu-Leu-leucinal (MG132) were obtained from Calbiochem (Paisley, UK). I942 (*N*-(2,4-dimethylbenzenesulfonyl)-2-(naphthalen-2-yloxy)acetamide) was purchased from MolPort (Riga, Latvia). Recombinant human interleukin 6 (IL6) protein and recombinant human soluble IL6 receptor α (sIL6Rα) proteins were purchased from R and D Systems (Abingdon, UK).

### 2.2. Cell Culture and mRNA Extraction

HUVECs were grown in EGM2 at 37 °C and 5% (*v*/*v*) CO_2_. Cells were passaged weekly to a maximum of six passages. HUVECs were grown on 6-well plates until they had achieved 70–80% confluence. Cells were then incubated in the presence or absence of 100 μM I942 or 10 μM forskolin and 10 μM rolipram (F/R) for 48 h. Total RNA was then isolated from cells using an RNeasy Kit (Qiagen, Manchester, UK), according to the manufacturer’s protocol. RNA concentration was determined using NanoDrop Spectrophotometer (Thermo Fisher Scientific, Paisley, UK). Isolated total RNA was used for RNA sequencing and quantitative Real-Time PCR analysis. 

### 2.3. RNA Sequencing (RNA-SEQ)

Sequencing libraries were prepared from total RNA using the Illumina TruSeq Stranded mRNA Sample Preparation Kit. Libraries were sequenced in 75 base, paired end mode on the Illumina NextSeq 500 platform. Raw sequence reads were trimmed for contaminating sequence adapters and poor quality bases using the program Cutadapt1 [17]. Bases with an average Phred score lower than 28 were trimmed. Reads that were trimmed to less than 54 bases were discarded. The quality of the reads was checked using the Fastqc program [18] before and after trimming. The reads were “pseudo aligned” to the transcriptome using the program Kallisto2 [19]. The differential expressions for the analysis groups were assessed using the Bioconductor package DESeq2 [20]. Duplicate results were removed from the resulting gene lists and fold changes and adjusted p-values were log-transformed. Custom scripts for MATLAB were written to create volcano plots and to compare gene lists for both treatments.

### 2.4. Chromatin Immunoprecipitation and Sequencing (ChIP-SEQ) Analysis

HUVECs were stimulated in the presence or absence of 100 μM I942 or F/R for 48 h at 37 °C in 5% (*v*/*v*) CO_2_. Cells were then fixed and chromatin was extracted and sheared using the enzymatic “CHIP-IT Express Kit” (Active Motif) according to the manufacturer’s instructions. Sheared chromatin from I942-treated, F/R-treated and non-treated cells was then immunoprecipitated at 4 °C, overnight with 4 μg of either C/EBPβ or c-Jun ChIP-grade antibodies (Santa Cruz). DNA fragments were eluted from immunoprecipitated chromatin and used to prepare a ChIP-SEQ DNA library for sequencing using the “ChIP-SEQ Sample Prep Kit” from Illumina, according to the manufacturer’s protocols. Briefly, the first step in library preparation was to convert any overhangs in the ChIP’d DNA into phosphorylated blunt ends. The 3′ ends were then adenylated and adaptors ligated onto the ends of the fragments. The library was then size selected on an agarose gel and eventually enriched by PCR. The enriched library samples were then loaded onto a flow cell at a concentration of 12 pM and cluster formation was done on an Illumina Cluster station. Samples were then sequenced on an Illumina GA IIX giving 76 bp reads.

### 2.5. ChIP-SEQ Data Analysis

The ChIP DNA was sequenced on an Illumina GA IIx, one lane of the flow cell per sample. The quality of the reads was assessed using Fastqc [18]. The sequence reads were aligned to the mouse genome (release version mm9) using the Bowtie aligner (version 0.12.7) [21,22], which was set up to report only uniquely aligning reads. Duplicate reads were removed using Samtools (version 0.1.18) [22]. The ChIP analysis was performed using the Homer (version 3.9) suite of tools [23]. Custom scripts were created in MATLAB to compare the list of known genes closest to ChIP peaks with gene lists generated from RNA-SEQ experiments.

### 2.6. Quantitative Real-Time PCR

HUVECs were stimulated in the presence or absence of 100 μM I942 or a combination of 10 μM forskolin plus 10 μM rolipram (F/R) for 48 h at 37 °C in 5% (*v*/*v*) CO_2_. For reverse-transcription, 1 μg of total RNA was converted to first-strand cDNA using RT^2^ First Strand Kit (Qiagen) in accordance with the manufacturer’s instructions. First, genomic DNA was eliminated using the buffer provided in the kit (5 min at 42 °C), followed by reverse-transcription reaction (15 min at 42 °C followed by 5 min at 95 °C). Real-Time PCR analysis was performed using the Qiagen Human Endothelial Cell Biology RT2 Profiler PCR Array (384-well format containing 4 × 96 PCR arrays) and RT^2^ SYBR Green Mastermix (Qiagen) using a 7900HT Fast Real-Time PCR System (Thermo Fisher Scientific), according to the manufacturer’s protocol. Each PCR Array included 89 validated qPCR Primers Assays, including 5 housekeeping genes and a control panel. The thermal cycling program was as follows: 10 min at 95 °C for HotStart DNA Taq Polymerase activation, 40 cycles of denaturation at 95 °C for 15 s and annealing and extension at 60 °C for 1 min. Each experiment was run in triplicate. Data analysis and quantification of relative mRNA gene expression were performed by the CT method using the free PCR Array Data Analysis web portal [24]. 

### 2.7. siRNA Procedures

For siRNA treatments, cells were seeded at 70,000–100,000 cells per well of a 6-well culture plate and grown in 2 mL complete growth medium until 50–60% confluent. The c-Jun EPAC1 siRNA (Qiagen FlexiTube Hs_RAPGEF3_5) or non-targeting siRNA (Qiagen AllStars Negative Control) was prepared for transfection by mixing 7 µL PromoFectin-HUVEC solution (Promocell) in 100 µL serum-free culture medium with 12 µL siRNA (from 20 µM stock), also dissolved in 100 µL serum-free medium, and then incubating for 20 min at room temperature. Following this, the cell culture medium was replaced with 0.9 mL fresh serum-free medium and the siRNA/PromoFectin-HUVEC solution (200 µL) was added dropwise, while gently shaking the plate, giving a final siRNA concentration of 200 nM. Cells were then incubated at 37 °C in 5% (*v*/*v*) CO_2_ for 4 h. After incubation, the medium was removed carefully by aspiration and then 2 mL of fresh complete growth medium was added, and cells were further incubated for 48 h, after which experimental procedures were carried out.

### 2.8. Western Blotting

For Western blotting, cells were harvested by scraping directly into 150 μL of SDS-polyacrylamide gel electrophoresis sample buffer [62.5 mM Tris-HCl, pH 6.8, 2% (*w*/*v*) SDS, 10% (*v*/*v*) glycerol, 10 mM DTT, and 0.01% (*w*/*v*) bromophenol blue]. Samples were mixed by vortexing, denatured by heating for 5 min at 95 °C, separated on 10% (*w*/*v*) resolving gels and then electroblotted onto nitrocellulose membranes. Membranes were then blocked in 5% (*w*/*v*) milk powder in Tris-buffered saline containing 0.1% (*v*/*v*) Tween 20. Blots were incubated in primary antibodies overnight at 4 °C followed by appropriate horseradish peroxidase-conjugated secondary antibodies for 1 h at room temperature. Blots were then developed using SuperSignal™ West Pico Chemiluminescent Substrate and visualised using a Fusion FX7-SPECTRA system (Vilber, Germany) fitted with a CCD camera.

### 2.9. Densitometry and Statistical Analysis

Non-saturated immunoblots from multiple experiments were quantified densitometrically using ImageJ software [25]. Statistical significance was determined by one-way ANOVA using InStat Software (GraphPad Software, San Diego, CA, USA).

## 3. Results

### 3.1. Identification of Genes Regulated by Cyclic AMP and I942 in HUVECs

We previously showed that when activated by cyclic AMP, the EPAC1/Rap1 pathway mediates the induction of the SOCS3 gene in human umbilical vein endothelial cells (HUVECs), independently of PKA activation [8,9], and that EPAC1 activation by I942 has the potential to suppress IL6 pro-inflammatory gene expression through the inhibition of JAK/STAT3 signalling in the same cells [26].

**Figure 1:**
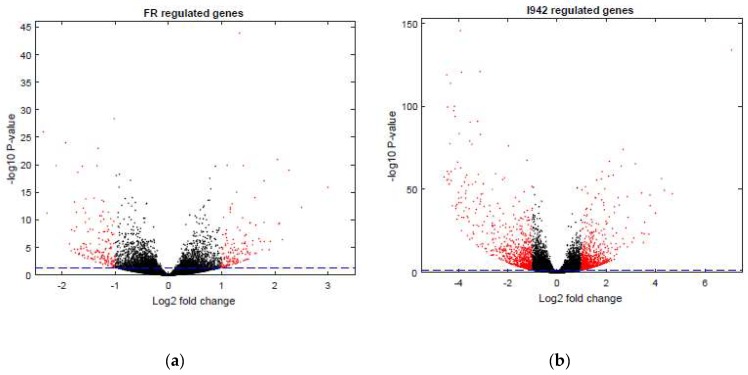

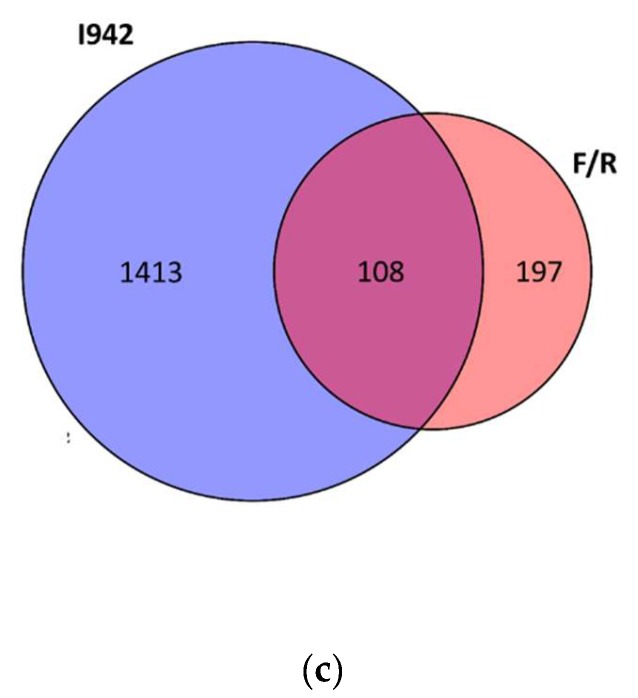
In order to identify genes regulated by I942, confluent human umbilical vein endothelial cells HUVECs were stimulated for 48 h with (**a**) 10 μM forskolin plus 10 μM rolipram (F/R) or (**b**) 100 μM I942. Total RNA was then extracted from cells and processed for RNA-SEQ and plotted as volcano plots as described in Materials and Methods, with gene expression changes greater than 2-fold being indicated in red. Genes that were significantly (*p* < 0.05, dotted blue line) regulated individually or by both treatments (see Supplementary Materials) are expressed as a Venn diagram in (**c**).

**Figure 2:**
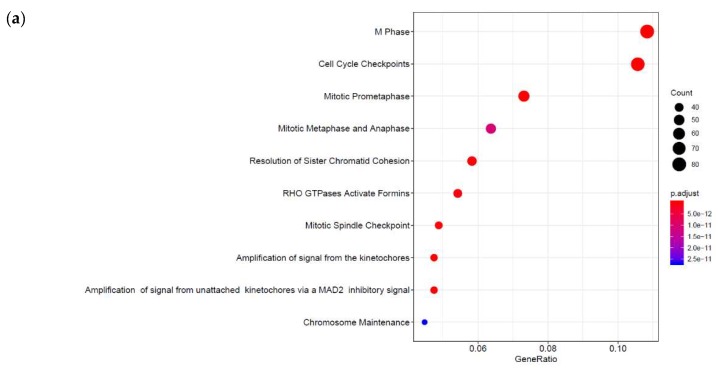

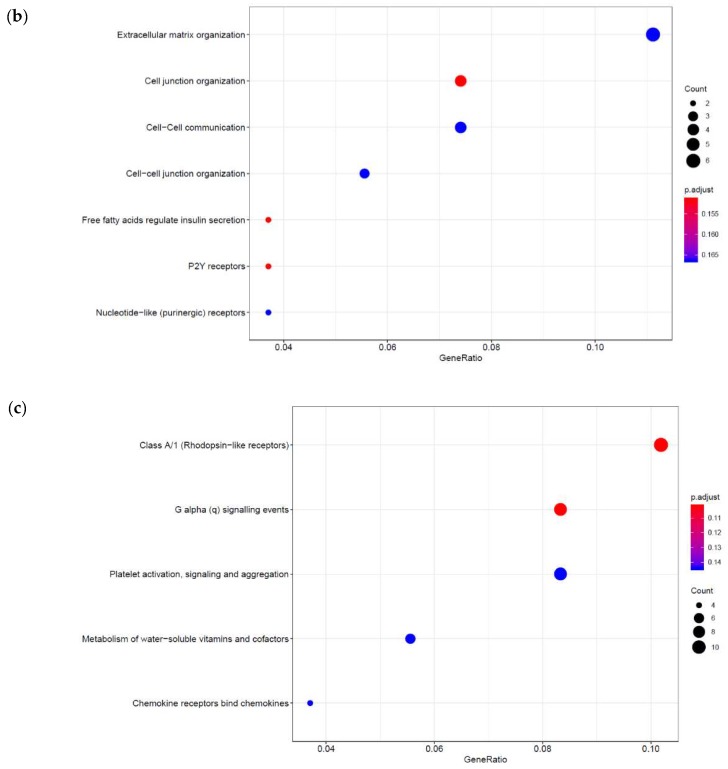
The data forming the Venn data in Figure 1 were analysed by the Bioconductor ReactomePA software package [30] to generate gene ontology (GO; *y*-axis) maps for significant F/R (**a**), I942 (**b**) and shared (**c**) genes. The *x*-axis represents the ratio of genes in the ontology over the total number of enriched genes. The occurrence of individual gene ontologies (count) and significance (*p*-adjust) are also displayed, as indicated in the key on the right of the graphs.

However, we still had to determine the full range of genes regulated by cyclic AMP and I942 in VECs. Here we aimed to identify genes in HUVECs regulated by elevations in global cyclic AMP, as induced by a combination of the adenylyl cyclase activator, forskolin, and the type 4 PDE inhibitor, rolipram (F/R), and further determine their responsiveness to I942 treatment. We therefore performed RNA-sequencing (RNA-SEQ) in HUVECs treated for 48 h with either I942 or F/R (Supplementary Data File). Volcano plots from these mRNA reads (Figure 1a,b) demonstrate that both treatments induced a profound up-regulation or down-regulation of target genes, many of which were greater than 2-fold in magnitude (indicated in red in Figure 1a,b). Further analysis identified 108 genes whose activities were similarly regulated by both I942 and F/R treatments, whereas 1413 gene expression changes were attributable to I942 treatment alone and 197 gene expression changes were attributable to F/R treatment alone (Venn diagram in Figure 1c). Gene ontology (GO) analysis of these gene expression changes indicates that I942 predominantly regulates genes associated with microtubule functions associated with chromosome functions and cell cycle progression (Figure 2, middle panel). Genes regulated by F/R included those associated with specific VEC functions (Figure 2, upper panel), including cell junction organisation and purinergic signalling, which is associated with anti-inflammatory actions in these cells [4,27,28,29]. Gene expression changes shared by both treatments also included those normally associated with specific VEC cell functions, including inflammatory actions and platelet activation (Figure 2, lower panel).

### 3.2. Genome-Wide Mobilisation of c-Jun and C/EBPβ Transcription Factors by Cyclic AMP and I942

**Figure 3:**
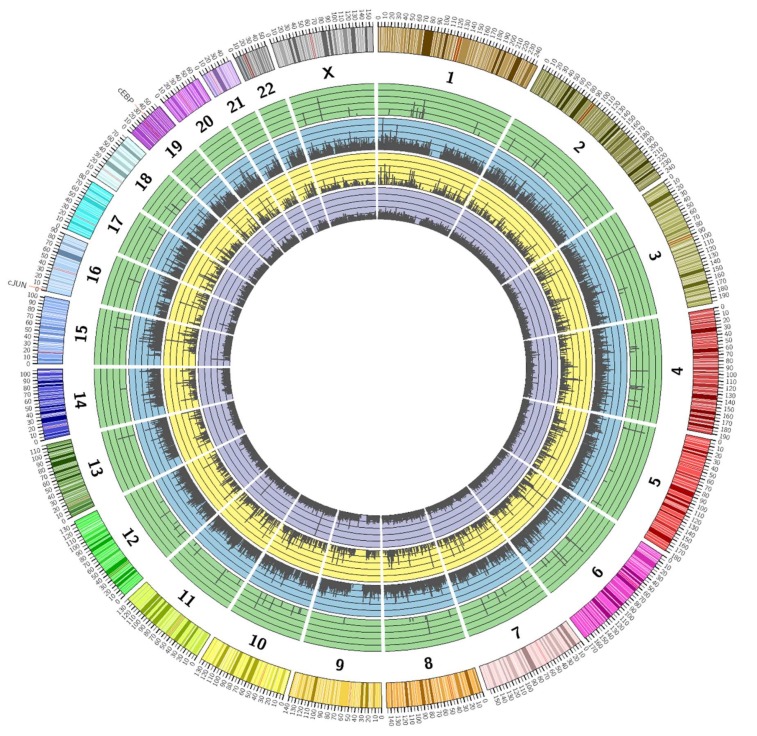
Custom scripts were created to compare the list of known genes closest to ChIP peaks (see Supplementary Materials). These corroborated gene lists were visualised on a chromosome map using Circos plots (circos.ca). Genes that demonstrate c-Jun binding following stimulation are indicated by peaks in the purple (for I942) and yellow (for F/R) coloured bands, whereas genes that demonstrate C/EBPβ binding sites following stimulation are in the blue (for F/R) and green (for I942) coloured bands. Chromosome number is indicated by numerals in bold and chromosome positions are indicated in the outside ring.

Using the paradigm of induction of the SOCS3 gene by cyclic AMP, we previously showed that non-canonical regulation of gene transcription following EPAC1 activation in HUVECs is associated with the activation and chromatin recruitment of c-Jun and C/EBPβ transcription factors [9,31]. Here we extended these studies to determine if the gene expression changes associated with F/R and I942 treatment in Figure 1 are also associated with recruitment of these transcription factors to regulated gene promoters. For this, we applied chromosome precipitation followed by next generation sequencing (ChIP-SEQ) to identify transcription factor binding sites for F/R and I942-regulated c-Jun and C/EBPβ throughout the human genome. For this, HUVECs were stimulated for 48 h with either F/R or I942, then cells were fixed, lysed and cellular chromatin was isolated, fragmented and immunoprecipitated (ChIP’d) with anti- c-Jun or anti-C/EBPβ antibodies. The genomic DNA associated with ChIP’d samples was then sequenced using a genome analyser to identify binding sites for c-Jun and C/EBPβ throughout the human genome. The HOMER suit of in silico tools was used to verify that a significant majority of genes contained bona fide c-Jun and C/EBPβ DNA binding sites (Table 1). While this was found to be the case, it was observed that CEBPβ activated by I942 was found to associate significantly with CREB binding sites (Table 1), which might mirror a level of cross-reactivity between cyclic AMP-activated transcription factors at the genome level. Further analysis using a Circos plot to map c-Jun and C/EBPβ binding to known genes demonstrates that treatment with either F/R or I942 promotes genome wide association of transcription factors, with binding sites on all chromosomes (Figure 3). It should be noted that treatment with F/R promoted more extensive genome interactions with c-Jun and C/EBPβ than those promoted by I942 treatment (Figure 3), suggesting that cooperation between PKA and EPAC signalling might be required for full transcriptional activation.

**Figure 4:**
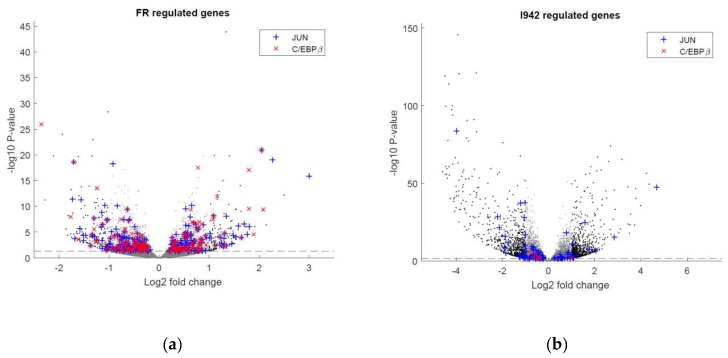
The RNAseq data generated for Figure 1 was re-analysed to identify gene expression changes associated with c-Jun (blue) or C/EBPβ (red) recruitment associated with either (**a**) F/R or (**b**) I942 treatment from ChIP-SEQ experiments.

While the Circos plot in Figure 3 accurately maps DNA binding sites throughout the human genome, it does not indicate whether transcription factor recruitment leads to induction or suppression of target gene expression. To examine this, and to complement the ChIP-SEQ experiments, the RNA-SEQ data presented in Figure 1 was re-analysed to determine whether the genes that contain c-Jun- or C/EBPβ-binding sites, identified by ChIP-SEQ, are also regulated at the level of transcription (Figure 4). 

It can be seen in Figure 4a that the majority of significant gene expression changes induced by F/R in HUVECs contain binding sites for active c-Jun and/or C/EBPβ. In contrast, genes regulated by I942 treatment contained very few C/EBPβ binding sites and instead favour genome interaction with c-Jun (Figure 4b). This suggests that c-Jun and C/EBPβ may play a minor role in the regulation of gene expression by I942 yet play a major role in gene responsiveness to elevations in intracellular cyclic AMP.

**Figure 5:**
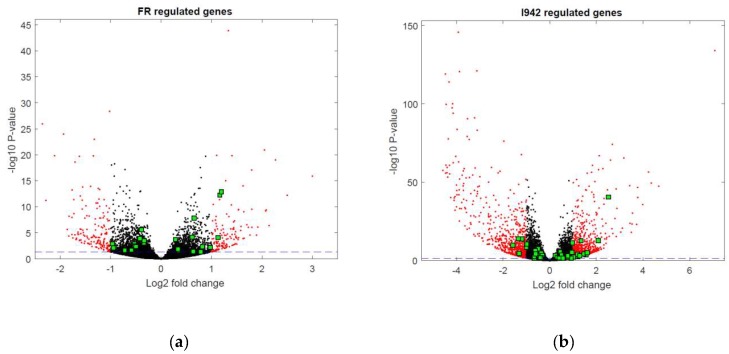
The RNA-SEQ data generated for Figure 1 was re-analysed to identify changes in the expression of 84 genes in (**a**) F/R and (**b**) I942 treated cells, which were previously identified as being specifically associated with endothelial cell function (green squares). Gene identities include those associated with angiogenesis, vasoconstriction/dilation, inflammation, apoptosis, cell adhesion, coagulation and platelet aggregation [35] and are subsequently used in Figure 6 for RT-PCR experiments.

**Figure 6:**
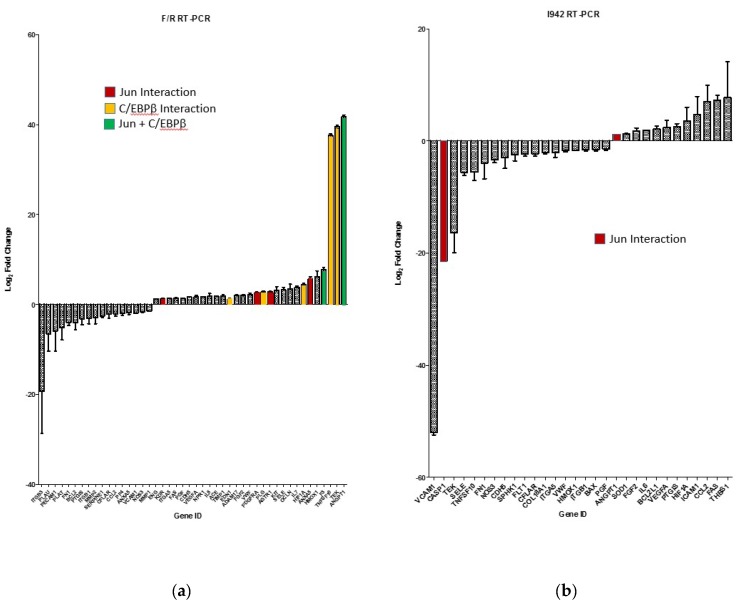

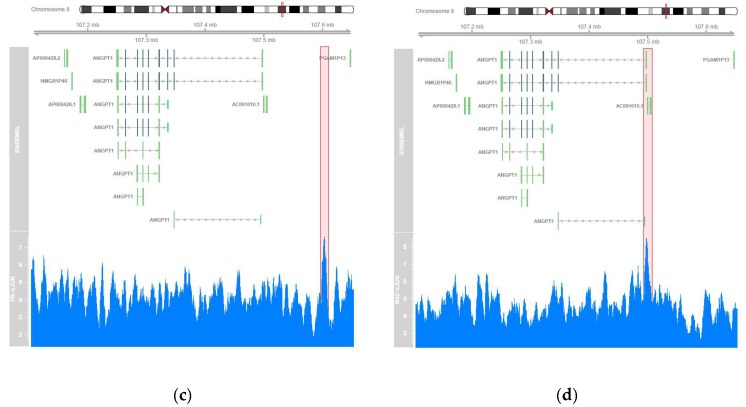
HUVECs were stimulated for 48 h with (**a**) F/R or (**b**) 100 μM I942 or and then total cell RNA was extracted and subjected to RT-PCR using a RT2 Profiler™ PCR Array for Human Endothelial Cell Biology as described in Materials and Methods. The bar graph is coloured to show genes that were identified by ChIP-SEQ as interacting with either C/EBPβ (yellow), c-Jun (red) or both C/EBPβ and c-Jun (green). Plotted in (**c**) and (**d**) is the region for ANGPT1, which interacts with c-Jun and is induced by both F/R (**a**) and I942 (**b**), along with the sequence read coverage (in blue) in that region from alignments. Stimulation with either F/R (**c**) or I942 (**d**) results in a peak immediately up-stream of the ANGPT1 gene, although the binding site appears different for each stimulus.

**Figure 7:**
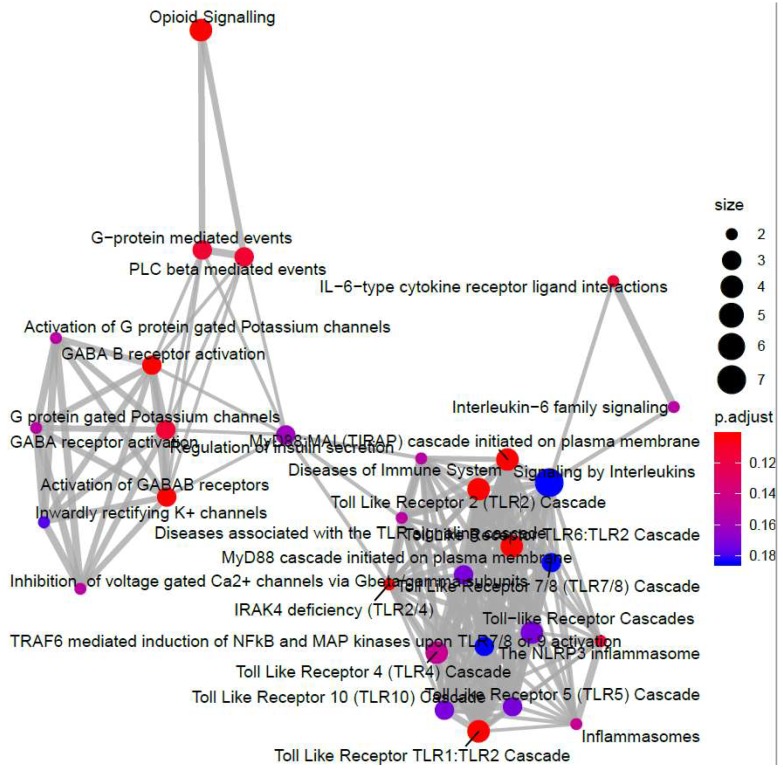
The RNA-SEQ data generated for Figure 4 was re-analysed using ReactomePA software to identify gene expression changes in I942-stimulated cells that were associated with interaction with c-Jun and are presented here as a CNET plot of genes with associated functions.

To examine this further, we re-examined RNA-SEQ data to determine whether activation of c-Jun and C/EBPβ is associated with the regulation of 84 genes known to be involved in endothelial cell function (Figure 5). These candidate genes are involved in functions such as inflammation, cell adhesion, platelet activation, angiogenesis, coagulation and apoptosis (Figure 5). We also used Human Endothelial Cell Biology RT^2^ Profiler™ PCR Arrays to examine the expression of the same set of 84 endothelial-specific genes in HUVEC cells following F/R and I942 treatment (Figure 6). We found that treatment of HUVECs with F/R or I942 for 48 h led to either an up- or down-regulation of endothelial-specific genes in HUVECs as determined by either RNA-SEQ experiments (Figure 5) or RT-PCR experiments (Figure 6). Importantly, by cross-referencing with ChIP-SEQ data, we found that many of the genes up regulated by F/R treatment were also associated with c-Jun and/or C/EBPβ interaction (Figure 6a), highlighting an important role for these transcription factors in the response to cyclic AMP in VECs. In contrast, we could find no binding of C/EBPβ to endothelial-specific genes regulated by I942, and only two genes interacting with c-Jun (Figure 6b). Of these two, induction of the ANGPT1 gene, is associated with c-Jun binding to upstream sequence elements, following stimulation with either F/R (Figure 6c) or I942 (Figure 6d). Despite this, certain key inflammatory genes, including IL6, VEGF, CCL2/MCP1, VCAM1, SELE and ICAM1 were regulated by I942, without significant c-Jun recruitment (Figure 6b), suggesting an indirect mode of action. Although I942 clearly promotes genome-wide mobilisation of c-Jun and C/EBPβ (Figure 3) it does not appear to play a direct role in the regulation of endothelial-specific genes by I942. Indeed, GO analysis of I942-regulated genes that interact with c-Jun (Figure 7) identify multiple gene families involved in receptor signalling, including the IL6 signalling pathway, which we have previously shown to be inhibited by I942 in HUVECs [34]. I942 may therefore regulate c-Jun-dependent transcription through the regulation of receptor signalling pathways including the IL6 pathway.

### 3.3. c-Jun Is Required for SOCS3 Induction and Suppression of IL6 Signalling by I942 in HUVECs

**Figure 8:**
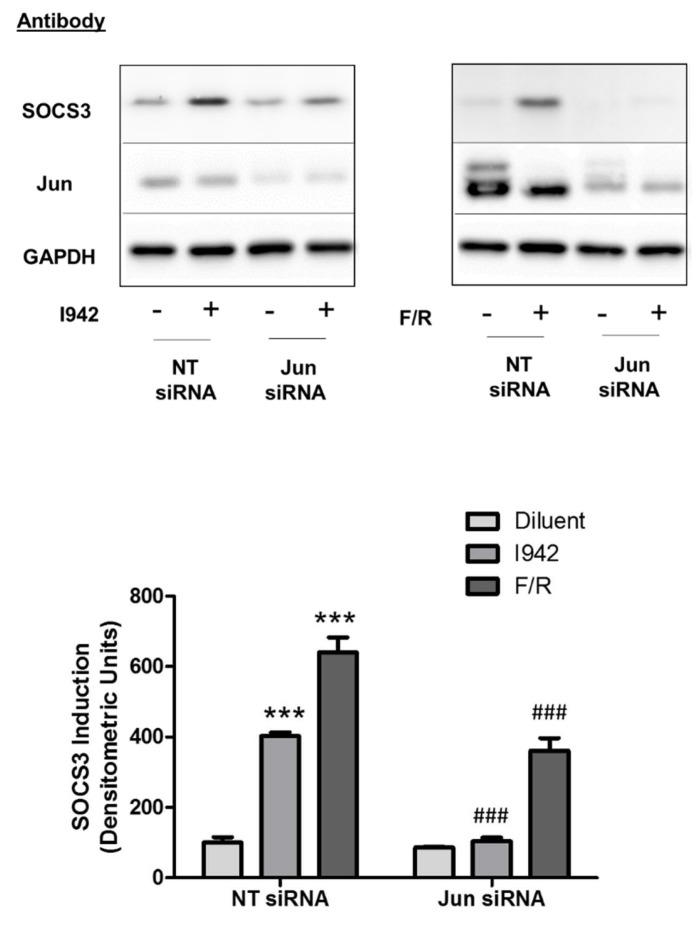
Confluent HUVECs were pre-incubated with siRNA to c-Jun or non-targeting siRNA (NT) for 24 h and then stimulated for 5 h in the presence or absence of 100 μM I942 (upper left panel) or 10 μM F/R (upper right panel). Cell extracts were then prepared and immunoblotted with antibodies to SOCS3 protein, c-Jun and GAPDH, as a loading control. Densitometry was carried out on 3 Western blots and results are shown as a bar graph. Significant increases in SOCS3 protein expression, relative to diluent-stimulated control cells, are indicated; *** *p* < 0.001. Significant inhibition of SOCS3 induction relative to NT siRNA-treated cells is also indicated; ### *p* < 0.001.

To confirm the GO analysis in Figure 7, which suggests I942 regulates IL6 signalling through an indirect, c-Jun-dependent mechanism we determined the impact of I942 on IL6 signalling in HUVECs. It has previously been shown that EPAC1 activation in HUVECs leads to SOCS3 gene induction and suppression of IL6 mediated STAT3 and ERK activation [4,11,34]. Moreover, we recently demonstrated that I942 can also induce SOCS3 expression to suppress STAT3 activation by IL6 in HUVECs [11]. We have also shown that SOCS3 induction by EPAC1 requires activation of c-Jun and C/EBPβ transcription factors, which interact with an essential AP1 transcription factor-binding site within the SOCS3 minimal promoter [12,31]. We therefore determined whether I942 and F/R, could also induce c-Jun-dependent SOCS3 expression in HUVECs (Figure 8). We found that depletion of c-Jun with siRNA significantly inhibited SOCS3 induction by both I942 and F/R (Figure 8). These results suggest that c-Jun is required for SOCS3 induction by I942 with the potential to inhibit IL6 signalling. 

**Figure 9:**
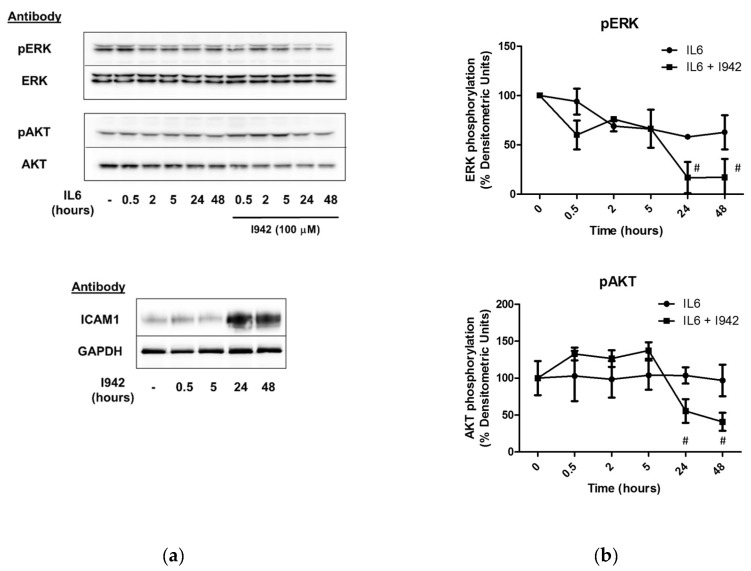
(**a**) HUVECs were stimulated for the indicated times with IL6 (5 ng/mL) plus sIL6Rα (25 ng/mL) in the presence or absence of 100 μM I942. Cell extracts were then immunoblotted with antibodies to pERK/ERK, pAKT/AKT, ICAM1 or GAPDH, as indicated. (**b**) Densitometric values from 3 separate immunoblots are shown on the left with significant decreases in the ratio of pERK/ERK and pAKT/AKT in cells stimulated with IL6 and I942, relative to stimulation with IL6 alone, being indicated; # *p* < 0.05 (*n* = 3).

In agreement with this, we show here that I942 also significantly inhibits late-stage ERK and AKT activation in response to IL6 signalling in HUVECs (Figure 9). One of the effects of long-term IL6 treatment is up-regulation of the protein product of the ICAM1 gene, as previously demonstrated [26], which correlates with inhibition of IL6-activated ERK, AKT (Figure 9) and STAT3 [26]. This suggests that induction of c-Jun-dependent SOCS3 has the ability to indirectly regulate ICAM1 gene expression through inhibition of IL6-regulated signalling pathways.

**Figure 10:**
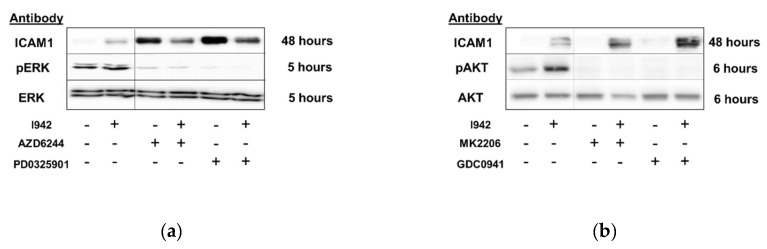

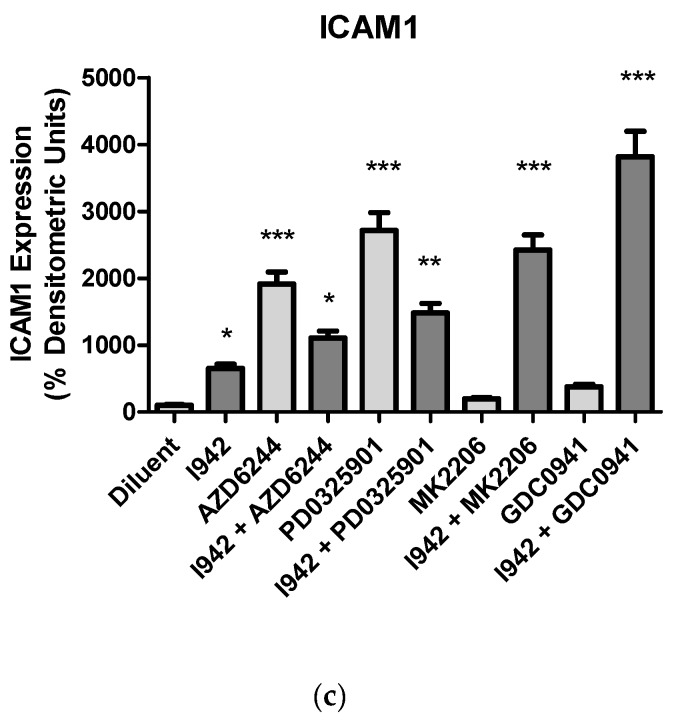
(**a**) HUVECs were stimulated for the indicated times with the MEK inhibitors, AZD6244 or PD032590, in the presence or absence of 100 μM I942. Cell extracts were then prepared and immunoblotted with anti-ERK and phospho-ERK antibodies or anti-ICAM1 antibodies, as indicated. (**b**) HUVECs were stimulated for the indicated times with the PI3 kinase inhibitor, GDC0941 (10 μM), or the AKT inhibitor, MK2206 (10 μM). Cell extracts were then immunoblotted with anti-AKT and phospho-AKT antibodies or anti-ICAM1 antibody. (**c**) ICAM1 densitometric values from 3 separate immunoblots are shown as a bar graph with significant changes in ICAM1 expression, relative to cells stimulated with diluent alone being indicated; * *p* < 0.05, ** *p* < 0.01, *** *p* < 0.001.

In agreement with this, we show here that I942 induces ICAM1 mRNA (Figure 6b and Supplementary Materials) and protein (Figure 9a) in HUVECs and this is further enhanced by inhibition of ERK and AKT with selective inhibitors (Figure 10). Accordingly, the ERK inhibitors AZD6244 and PD0325901 provoked a dramatic increase in basal ICAM1 protein expression, as well as enhancing I942-induced ICAM1 expression (Figure 10a). This indicates that inhibition of ERK activity by prolonged I942 stimulation may be linked to induction of ICAM1 gene expression. Moreover, inhibition of PI3K/AKT signalling with GDC094 and MK2206, respectively, also potentiated ICAM1 induction by I942 (Figure 10b). Together these results demonstrate that the induction of ICAM1 by long-term I942 treatment is linked to c-Jun-dependent SOCS3 induction and late-stage suppression of ERK and AKT activities in HUVECs.

## 4. Discussion

The aims of this study were to compare and contrast non-canonical transcriptional regulation by cyclic AMP and a newly discovered selective EPAC1 agonist, I942. This is important because classical transcriptional regulation by cyclic AMP is thought largely to occur through PKA-mediated activation of the ATF family of transcription factors, the archetypical member being CREB [2]. It is not widely appreciated that PKA-independent mechanisms of gene transcription also exist, but this is now an important consideration since the discovery of PKA-independent modes of cyclic AMP action through novel effectors, including EPAC and POPDC proteins [36], suggests alternative mechanisms exist. In this regard, we have previously identified 425 I942-regulated genes that were are also regulated by the EPAC1-selective cyclic AMP analogue, 007, the majority of which are involved in the control of key vascular functions, including the gene for the cell adhesion molecule, VCAM1 [26]. Both I942 and 007 inhibited IL6-induced expression of VCAM1 at the protein level and blocked VCAM1-dependent monocyte adhesion to HUVECs [26]. This highlights an important role for non-canonical, EPAC1-dependent signalling mechanisms involved in the control of key vascular functions linked to disease.

Here we expanded on this original study and used a combination of RNA-SEQ and ChIP-SEQ to determine global gene responses to I942, in comparison with cyclic AMP production promoted by F/R, in HUVECs. Consistent with our previous RNA-SEQ studies we found that 108 gene expression changes were promoted by both treatments and are linked to the regulation of vascular function, including purinergic receptor signalling and cell junction organization, which are linked to inflammatory responses in these cells. Since these responses are duplicated by both treatments, it can be argued that these represent EPAC1-regulated responses. F/R treatment also promoted gene expression changes in a further 197 genes, again linked to endothelial cell function, including chemokine production and platelet aggregation. These responses therefore represent either PKA-dependent responses or a result of simultaneous activation of both EPAC1 and PKA signalling pathways. Treatment with I942 alone provoked significant changes in the RNA expression of 1413 genes, which were largely associated with microtubule stability and cell cycle progression (Figure 2). While it can be argued that many of the gene expression changes evoked by I942 could represent “off-target” effects, it should be pointed out that EPAC1 activation has already been linked to microtubule stability [37,38,39,40], cell cycle progression [41,42,43,44,45,46,47,48] and physical interaction with microtubule cytoskeleton components [49,50,51,52,53], so these results are consistent with “on-target” EPAC1-dependent actions of I942. Moreover, it has been shown that EPAC1 and PKA signalling responses are often mutually dependent [48,54]. It could also be envisaged that PKA activation may suppress certain EPAC1-dependent actions. The result of this kind of mutual dependency would result in EPAC1 activation by I942 provoking radically different cell responses to F/R treatment, which activates both EPAC1 and PKA. Further work will therefore be required to determine the interplay between EPAC1 and PKA signalling at the level of transcriptional control in VECs.

ChIP-SEQ analysis demonstrated that F/R treatment led to genome-wide recruitment of C/EBPβ and c-Jun transcription factors, whereas I942 promoted recruitment of c-Jun to genes associated with IL6 signalling and signalling from other receptor types, with little effect on C/EBPβ activity. In the case of F/R treatment, genome-recruitment of C/EBPβ and c-Jun was associated with the transcriptional regulation of the majority of genes identified by RNA-SEQ as being cyclic AMP sensitive (Figure 4a). This points towards a role for C/EBPβ and c-Jun as key players in non-canonical cyclic AMP signalling, although their mode of action remains to be determined. In this regard, we have previously shown that SOCS3 induction by EPAC1 in HUVECs requires the expression of protein kinase C (PKC) isoforms, α and δ [8,12]. However, it is not yet clear how these contribute to the regulation of C/EBPβ activity. We do know that PKCα and PKCδ are required for ERK activation by cyclic AMP, independently of EPAC1, leading to C/EBPβ activation through phosphorylation of Thr235 [8,12]. This would go some way to explain why I942 treatment does not lead to global recruitment of C/EBPβ in the present study, because direct activation of EPAC1 does not lead to ERK activation and hence phosphorylation of C/EBPβ on Thr235 [8]. Indeed, we show here that short-term stimulation of HUVECs has no effect on ERK activation (Figure 10a) and long-term stimulation actually inhibits IL6-promoted ERK activation (Figure 9b) and is therefore unlikely to activate C/EBPβ.

The mechanisms by which I942 regulates c-Jun activity also remain to be determined, but it has been known for some time that PKC enhances c-Jun activation by promoting the dephosphorylation of three key residues in the c-Jun DNA binding domain [55]. Indeed, mutation of one of these sites (Ser243) to phenylalanine inhibits the phosphorylation of all three sites, leading to enhanced c-Jun DNA binding activity [55]. It is therefore likely that I942, acting through EPAC1, leads to c-Jun activation through protein kinase C, including activation of phosphatases that target Ser243 of c-Jun. However, despite the ability of I942 to promote c-Jun DNA-binding activity, we found that key inflammatory genes, including IL6, VEGF, CCL2/MCP1, VCAM1, SELE and ICAM1 were regulated by I942, without significant c-Jun recruitment (Figure 6b), suggesting an indirect mode of action involving induction of SOCS3 and suppression of IL6-promoted ERK, AKT (Figure 9b) and STAT3 activation [26]. This would be brought about by SOCS3 binding to JAK-phosphorylated receptors, via the SOCS3 SH2 domain, thereby inhibiting JAK activity and, consequently, activation of STATs 1 and 3 [56], ERK and AKT [11]. SOCS3 would then also target multiple SH2-bound proteins for proteasomal degradation [56] with proteolytic targets including JAK2 [57]. IL6 has been reported to promote acute and chronic inflammatory disease in the absence of SOCS3 [58] and conditional deletion of the SOCS3 gene in VECs results in pathological angiogenesis [59]. Given this, novel treatments based on the regulation of SOCS3 levels in cells, as shown for I942 here, could therefore have efficacy in the treatment of inflammatory diseases where there is over-stimulation of JAK signalling. 

## 5. Conclusions

**Figure 11:**
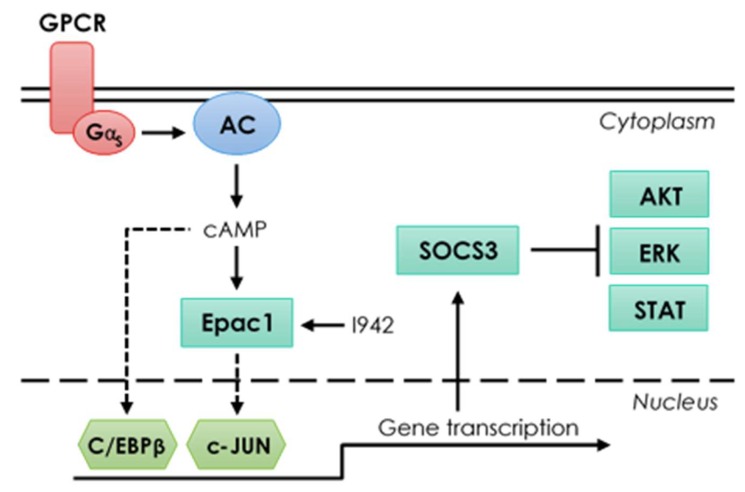
Summary of signalling mechanisms in the present study.

Transcriptional regulation of endothelial-specific genes by cyclic AMP is associated with genome-wide recruitment of c-Jun and C/EBPβ transcription factors, which are activated through non-canonical signalling mechanisms. The novel EPAC1 activator, I942, also regulates endothelial-specific gene expression through distinct mechanisms involving either direct recruitment of c-Jun to target gene promoters, as is the case for SOCS3, or through indirect regulation of tertiary regulators, including SOCS3, which inhibit gene-regulatory signalling pathways, including IL6-activated ERK, AKT and STAT3 (please see Figure 11 for graphical summary).

## Figures and Tables

**Table 1:** Results of c-Jun and C/EBPβ ChIP-SEQ. Human umbilical vein endothelial cells (HUVECs) were incubated for 48 h in the presence or absence of I942 or forskolin and rolipram (F/R). Following stimulation cells were fixed, chromatin was isolated and then immunoprecipitated (ChIP’d) with anti-c-Jun or anti-C/EBPβ antibodies, as described in Materials and Methods. ChIP’d DNA samples were then sequenced (ChIP-SEQ) on an Illumina GA IIx DNA sequencer. ChIP analysis of the resulting DNA sequences was then performed using the Homer (version 3.9) suite of tools [23]. The figure shows part of the Homer analysis indicating that aligned sequences from each ChIP experiment contained bone fide AP-1 [32] and C/EBP [33] consensus binding motifs, hence validating the experimental technique.

ChIP	Treatment	Motif	Alignment	Name	*p*-Value
C/EBPβ	F/R	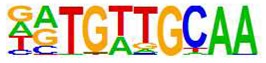	RTGTTGCAA--TTNNNCAAY	CEBP/AP1	0.001
C/EBPβ	I942	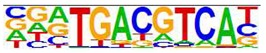	TGACGTCATGACGTCA	CREB/ATF	0.001
c-Jun	F/R	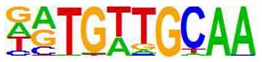	RTGTTGCAA--TTNNNCAAY	CEBP/AP1	0.001
c-Jun	I942	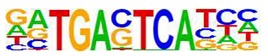	TGASTCATGAGTCA	Jun/AP1	0.001

## Supplementary Materials

The following are available online at https://www.mdpi.com/2073-4409/8/10/1253/s1. A supplementary data file detailing all SEQ data for each figure accompanies this manuscript.
